# Claims-Based Algorithm to Identify Pre-Exposure Prophylaxis Indications for Tenofovir Disoproxil Fumarate and Emtricitabine Prescriptions (2012-2014): Validation Study

**DOI:** 10.2196/55614

**Published:** 2024-11-04

**Authors:** Patrick Sean Sullivan, Robertino M Mera-Giler, Staci Bush, Valentina Shvachko, Eleanor Sarkodie, Daniel O'Farrell, Stephanie Dubose, David Magnuson

**Affiliations:** 1 Department of Epidemiology Rollins School of Public Health Emory University Atlanta, GA United States; 2 Universidad Espiritu Santo Guayaquil Ecuador; 3 Brooklyn, NY United States; 4 Gilead Sciences Foster City, CA United States; 5 Whitman Walker Institute Washington, DC United States

**Keywords:** pre-exposure prophylaxis, PrEP, classification, electronic medical record, EMR, algorithm, electronic health record, EHR, drug, pharmacology, pharmacotherapy, pharmaceutical, medication, monotherapy, HIV, prevention

## Abstract

**Background:**

To monitor the use of tenofovir disoproxil fumarate and emtricitabine (TDF/FTC) and related medicines for pre-exposure prophylaxis (PrEP) as HIV prevention using commercial pharmacy data, it is necessary to determine whether TDF/FTC prescriptions are used for PrEP or for some other clinical indication.

**Objective:**

This study aimed to validate an algorithm to distinguish the use of TDF/FTC for HIV prevention or infectious disease treatment.

**Methods:**

An algorithm was developed to identify whether TDF/FTC prescriptions were for PrEP or for other indications from large-scale administrative databases. The algorithm identifies TDF/FTC prescriptions and then excludes patients with *International Classification of Diseases (ICD)–9* diagnostic codes, medications, or procedures that suggest indications other than for PrEP (eg, documentation of HIV infection, chronic hepatitis B, or use of TDF/FTC for postexposure prophylaxis). For evaluation, we collected data by clinician assessment of medical records for patients with TDF/FTC prescriptions and compared the assessed indication identified by the clinician review with the assessed indication identified by the algorithm. The algorithm was then applied and evaluated in a large, urban, community-based sexual health clinic.

**Results:**

The PrEP algorithm demonstrated high sensitivity and moderate specificity (99.6% and 49.6%) in the electronic medical record database and high sensitivity and specificity (99% and 87%) in data from the urban community health clinic.

**Conclusions:**

The PrEP algorithm classified the indication for PrEP in most patients treated with TDF/FTC with sufficient accuracy to be useful for surveillance purposes. The methods described can serve as a basis for developing a robust and evolving case definition for antiretroviral prescriptions for HIV prevention purposes.

## Introduction

Pre-exposure prophylaxis (PrEP) with tenofovir disoproxil fumarate and emtricitabine (TDF/FTC) is used for HIV prevention and is a mainstream pillar of comprehensive HIV prevention programs [1-3]. The National HIV/AIDS Strategy established national targets for PrEP used in 2015, calling for a 500% increase in PrEP use between 2015 and 2020 [4]. These targets have been challenging to measure rigorously; specifically, measurement of uses of antiretroviral therapies (specifically, use of TDF/FTC, the only US Food and Drug Administration (FDA)–approved medication for PrEP from 2015 to 2019) has been far from straightforward. TDF/FTC is a medication that is also used for HIV treatment in combination with other antiretroviral medications, can also be used in regimens for postexposure prophylaxis (PEP), and is sometimes prescribed off-label as for the management of chronic hepatitis B (CHB). For these reasons, to monitor the uptake of TDF/FTC for PrEP indication using data from prescription databases, we must first identify apparent prescriptions for TDF/FTC and then rule out prescriptions that were likely made for indications other than PrEP.

Considering methods to identify PrEP indications for TDF/FTC prescriptions relates to a larger trend in the use of prescription data to measure the use of prescription drugs in populations. Large health care use claims databases and electronic medical records (EMRs) are being increasingly used in pharmacoepidemiology studies [5-7]. There are limitations to such approaches because health care use claims data are primarily used for billing and do not record specific indications. To predict the intended indication for medications like TDF/FTC, for which multiple indications exist, validated algorithms can be used to identify a specific indication with sufficient confidence for use in surveillance. In these algorithms, additional data related to the prescription, such as patient characteristics of interest in the form of codes, procedures, or laboratory results, can help to predict the intended indication. Algorithms using claims data should be developed and validated to support their use for monitoring the use of prescription drugs in populations. Because individuals prescribed TDF/FTC can be identified through commercial claims databases but PrEP indications are not directly available from these databases, we aimed to develop and validate an algorithm of exclusion criteria based on diagnosis indicators.

## Methods

### PrEP Indication Algorithm

The algorithm developed and evaluated uses prescription, diagnosis, and medical procedure data from administrative claims databases and EMRs to identify patients likely prescribed TDF/FTC for PrEP, by identifying TDF/FTC therapy that is not indicated for HIV treatment, CHB treatment, or HIV PEP. At the first stage, all patients prescribed TDF/FTC are enumerated and deduplicated. In the second stage, the algorithm excludes (1) patients previously diagnosed or treated for HIV, (2) patients previously diagnosed or treated for CHB, and (3) patients prescribed medications for HIV PEP. The algorithm is operationalized sequentially, so that the categories are not mutually exclusive (eg, a patient can be identified as being diagnosed with both HIV and CHB or can be diagnosed with CHB and prescribed HIV PEP). The algorithm evaluates each period of TDF/FTC prescription separately, such that a patient may have multiple exposure periods to TDF/FTC with different indications (eg, one exposure period for PrEP and a subsequent exposure period for treatment of HIV). The list of *International Classification of Diseases (ICD)–9* codes and antiretroviral medications used to define indications can be found in Table 1.

**Table 1:** *International Classification of Diseases (ICD)–9* diagnosis and generic product identifier medication codes used in an algorithm to identify likely uses of antiretroviral medications for HIV prevention indications, United States, 2012-2013.

Code			Description
***ICD-9***			
	042	A prior diagnosis of HIV disease	
	V08	Asymptomatic HIV infection	
	079.53	HIV-2 infection	
	112.84	Candidiasis of bronchi, trachea, or esophagus	
	112.4	Candidiasis of lungs	
	130.*	Toxoplasmosis	
	114.X	Coccidioidomycosis	
	117.5	Cryptococcosis	
	007.4	Cryptosporidiosis	
	078.5	CMV^a^ retinitis	
	176.X	Kaposi’s sarcoma	
	031.2, 031.0	Mycobacterium avium complex	
	136.3	Pneumocystis carinii pneumonia	
	70.22, 70.23, 70.32, 70.33	Chronic hepatitis B infection	
	E920.5	Contaminated needle stick	
	V078, V079	Prophylaxis	
**GPI^b^**			
	1210990230	FTC^c^/TDF^d^	
	121060300	FTC	
	1210857010	TDF	
	1210xxx	Other antiretroviral medications.	
	123520xxx	Hepatitis B agents: adefovir, entecavir, lamivudine, telbivudine	

^a^CMV: cytomegalovirus.

^b^GPI: generic product identifier.

^c^FTC: emtricitabine.

^d^TDF: tenofovir disoproxil fumarate.

A period of TDF/FTC exposure was defined as a continuous prescription of TDF/FTC (one prescription or multiple prescriptions where the gap between the end of the supply of one prescription and the start of the next prescription was <30 days). For each period, the index of the algorithm is as follows:

Among all exposure eras that belong to FTC/TDF, FTC, or TDF, sequentially:

1. Exclude all HIV condition eras where there is a previous diagnosis of HIV disease, asymptomatic HIV infection, HIV-2 infection, nonspecific serologic evidence of HIV, or a previous diagnosis of an opportunistic infection;
2. Exclude all exposure eras where there is a generic product identifier code for an antiretroviral, but retain FTC/TDF, FTC, or TDF;
3. Exclude all condition eras where there is a previous diagnosis of CHB infection;
4. Exclude all exposure eras where there are hepatitis B agents;
5. Exclude all condition eras with specific codes for contaminated needle stick or prophylaxis;
6. Exclude all condition eras not containing a diagnosis code (*ICD-9*) or a current procedural terminology code.

FTC/TDF eras that remain are divided into 2 categories:

1. Diagnosis and procedures availability (patient has health encounters with health care provider and had at least 1 procedure and 1 diagnosis included in the database): this group is considered as PrEP if taking FTC/TDF.
2. Availability of medications only: this group is not considered as PrEP.

From the rest of the potential PrEP eras (not HIV positive, not on HIV treatment, not CHB or on CHB treatment, and not PEP), only those that have existing information (encounters) and therefore any entry in both the diagnosis and procedure tables will be considered as PrEP. Date was assigned as the start of the period of exposure. Each period of TDF/FTC exposure was then assessed according to the following criteria (in sequential order); prescription periods that met any of the criteria were considered to have been prescribed TDF/FTC for an indication other than PrEP.

1. Any indicator of HIV infection or opportunistic infections before the index date;
2. Any previous use of other antiretrovirals;
3. Any concomitant use of antiretrovirals;
4. Any previous diagnosis of CHB;
5. Any concurrent or previous use of CHB treatment agents;
6. Any periods of TDF/FTC exposure categorized as part of PEP, per a published PEP algorithm [8];
7. Any periods with no previous diagnoses or procedures associated with them (ie, if no diagnosis or procedure codes were available, then the prescription period was conservatively assumed not to be for PrEP).

### Validation Approaches

The algorithm was validated using both (1) electronic data from a cohort of patients from a large EMR system and (2) medical record abstraction data from all TDF/FTC-treated patients from a large urban sexual health clinic.

#### EMR Dataset

We used an electronic health care dataset (US Quintiles Practice Research) containing deidentified patient-level data, which included demographic information, diagnoses, past and present medications, procedures, physician orders, and comments in text fields and laboratory data from 2012 to 2013. A retrospective case series was used to assess the algorithm and consisted of every patient in the database exposed to TDF/FTC for any indication. A total of 10,645 patients from the EMR data were prescribed TDF/FTC on 13,671 exposure periods between January 1, 2012, and December 31, 2013, and were available for analysis. Of these, a total of 810 patients with 916 TDF/FTC exposure periods and 1433 prescriptions were selected for chart review.

**Figure 1:**
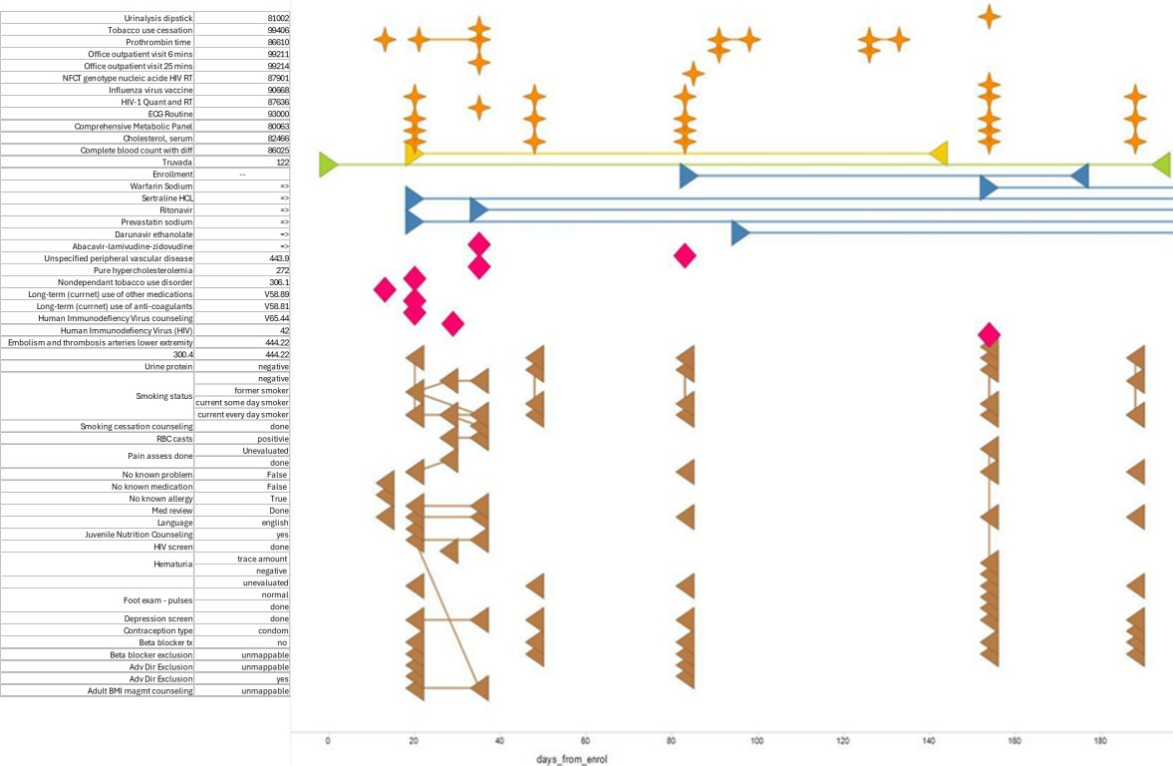
Example of a graphical patient profile used by clinicians to classify PrEP versus other uses in an evaluation of the performance of an algorithm to determine whether antiviral medications were used for HIV prevention, United States, 2012-2013. ECG: electrocardiogram; HCL: hydrochloride; NFCT: infection; PrEP: pre-exposure prophylaxis; RT: reverse transcriptase.

The algorithm validation methodology was operationalized in two steps: (1) the algorithm was applied to the EMR data tables and all patients with TDF/FTC therapy were assigned indications (eg, HIV treatment, CHB therapy, PEP, or PrEP) and (2) data from all patients identified by the algorithm as PEP, CHB, or PrEP and a 1% randomly selected sample of all patients identified as living with HIV were reviewed by 2 blinded investigators using a graphical patient profile containing all medications, diagnoses, procedures, comments in the medical record and laboratory tests on one screen (a deidentified sample is provided in Figure 1). The blinded investigators assigned proposed indications (eg, treatment vs PrEP) independently, and any mismatches were discussed and further evaluated by a third investigator (ES), who made the final determination. The chart review assignment of indication was considered the gold standard. The algorithm was also used to generate a predicted assignment of indication.

#### Large Community-Based Sexual Health Clinic

A large community-based clinic in Washington, District of Columbia, providing HIV care and serving >15,000 patients in care and 2200 patients prescribed PrEP under care participated in a clinic-based validation study and provided deidentified data from their EMRs. The clinic also separately provided a reference “gold standard list” of individuals who had been prescribed PrEP in 2012 through 2014. The gold standard list was developed by direct assessments from the health care providers who prescribed the medication. The validation methodology was applied to the EMR data tables for all patients exposed to TDF/FTC, a unique identifier was associated with the patient to protect privacy in the analyses, and indications (PrEP indication or not PrEP indication) were obtained from the algorithm. This list of patients was matched by the unique identifier to the “gold standard” list of patients known to have been using TDF/FTC for PrEP was provided by the clinic. Patients at the clinic who were known to have been prescribed TDF/FTC for PrEP by provider report comprised the gold standard list. By clinic administrative records, there were 2276 patients who were prescribed TDF/FTC between January 1, 2012, and December 31, 2014.

### Statistical Methods

For both studies, the primary outcome was agreement, as assessed by the κ statistic. A priori, we considered κ values of 0.41-0.60 to represent moderate agreement, values of 0.61-0.80 to indicate substantial agreement, and values above 0.80 to indicate almost perfect agreement [9,10]. Furthermore, the performance of the algorithm was characterized using a framework of diagnostic performance, in which the provider-derived indication was considered to be the gold standard and the algorithm as the new novel “test” for PrEP indication. In these analyses, the sensitivity, specificity, positive predictive value (PPV), and negative predictive value (NPV) of the algorithm were estimated; 2-sided CIs were calculated using Gaussian approximation as previously described [11]. Data were analyzed with Stata (StataCorp) and SAS Software (version 9.4; SAS Institute).

### Ethical Considerations

Data were collected in 2 settings. The commercial Quintiles dataset only contains deidentified patients’ information. We used the National Institutes of Health (NIH) Human Subjects Decision tool to document that the use of these evaluation of these data does not constitute human subjects research. For the evaluation of data from the large community-based sexual health clinic, institutional review board (IRB) approval was obtained before any review of medical records (Advarra IRB [IRB#00000971]). There was no interaction with human subjects, and there was no compensation of individuals for the secondary use of medical records data.

## Results

### EMR Validation Study

A total of 10,645 patients from the EMR data were prescribed TDF/FTC on 13,671 exposure periods between January 1, 2012, and December 31, 2013, and were available for analysis. Of these, 810 patients with 916 TDF/FTC exposure periods and 1433 prescriptions were selected for chart review. Based on EMR data alone, the algorithm identified 94.4% (12,905/13,671) of the total exposure periods as indications for treatment of HIV, 5.3% (724/13,671; further described below) as PrEP, 0.21% (29/13,671) as treatment for CHB, and 0.1% (13/13,671) as PEP. The study algorithm identified 671 patients as receiving PrEP (671/10,645, 6.3% of patients with TDF/FTC prescriptions). There was no instance where the algorithm identified a patient as having an HIV treatment indication based on ≥1 HIV-associated opportunistic infection who also did not have an HIV diagnostic code in the medical record. In the EMR data, the mean duration of exposure to TDF/FTC was 126 days for HIV infection, 239 days for CHB, 96 days for PrEP, and 26 days for PEP.

We compared the results of the algorithm-predicted indications for TDF/FTC with the clinician-determined indications (Table 2). The κ value was 0.53, indicating moderate agreement between the 2 methods [12]. When considering the clinician determination as the gold standard, the algorithm correctly classified 78.7% (721/916) of all exposure periods, with a sensitivity for the algorithm-assessed PrEP indication of 99.6% (95% CI 98.7%-100%) and a specificity for the algorithm-assessed non-PrEP indication of 49.6% (95% CI 44.5%-54.7%).

**Table 2:** Agreement of classification of tenofovir disoproxil fumarate and emtricitabine prescription periods as being for PrEP^a^ by computer algorithm of 916 PrEP potential prescription periods from a large electronic medical records database, United States, 2012-2013.

		Clinician assessment, n		Totals, n
		PrEP	Not PrEP	
**Algorithm assessment**				
	PrEP	531	193	724
	Not PrEP	2	190	192
**Totals **		533	383	916

^a^PrEP: pre-exposure prophylaxis.

The algorithm considered 724 exposure periods occurring in 671 patients to have a PrEP indication. The clinician-led chart review assigned 531 of those periods as PrEP, with a PPV for the algorithm-assigned PrEP indication of 73.3% (95% CI 71.4%-75.2%) and an NPV of 99% (95% CI 96.0%-99.7%). Considering the clinician-led chart review as the gold standard, the algorithm misclassified 4.6% (33/724) of all exposure periods as PrEP when they were truly CHB; 8.2% (59/724) as PrEP when they were truly for HIV treatment, and 9.3% (67/724) were classified as PrEP when they were truly PEP. For 4.7% (37/724) of all TDF/FTC periods classified as PrEP indications by the algorithm, the clinical review did not document enough evidence to substantiate the PrEP indication.

Based on the chart review, there was some additional information available on the misclassifications that occurred using the algorithm. We concluded that 9.3% (67/724) of TDF/FTC exposure periods on a patient who is HIV negative corresponded to high-risk sexual exposure PEP. This group of true PEP prescriptive was significantly associated with *ICD-9* codes such as V01.6, V01.79, and V01.9. *ICD-9* code V69.2 (high risk sexual behavior) was present in 23.2% (123/531) of all PrEP exposure periods but only in 0.67% (4/593) of HIV exposure periods and no CHB or PEP periods. V69.2 was 47.5 (95% CI 36.1-62.5) times more likely on PrEP periods than any other periods. Procedure codes, such as 99401, 99402, and 99403 (preventive medicine counseling and risk factor reduction interventions), were present in 29.4% (156/531) of all PrEP periods and were 33.2 (95% CI 25.2-43.8) times more likely to be present than in periods for other indications.

### Community-Based Clinic Validation Study

Among those prescribed TDF/FTC, 12% (275/2776) were reported by providers to have been prescribed TDF/FTC for a PrEP indication (Table 3). The algorithm correctly classified 88.4% (2013/2776) of all patients. The κ value for agreement was 0.61, indicating substantial agreement. The algorithm correctly identified 274 of the 275 “true” PrEP users (sensitivity 99.6%, 95% CI 98.0%-99.9%) and 1739 of the 2001 non-PrEP TDF/FTC users (specificity 87.1%, 95% CI 87.1%-89.8%). The PPV of an algorithm-designated PrEP user was 51.1% (95% CI 48.3%-53.9%) and the NPV of an algorithm-designated, non-PrEP user was 99.9% (95% CI 99.6%-99.9%).

**Table 3:** Agreement of classification of tenofovir disoproxil fumarate and emtricitabine prescriptions as being for PrEP^a^ by review of clinic records applied to data on 2276 patients prescribed tenofovir disoproxil fumarate and emtricitabine at a large community-based sexual health clinic, Washington, District of Columbia, 2012-2014.

		Clinic records (gold standard), n		Totals, n
		PrEP	Not PrEP	
**Algorithm assessment**				
	PrEP	274	262	536
	Not PrEP	1	1739	1740
**Totals**		275	2001	2276

^a^PrEP: pre-exposure prophylaxis.

## Discussion

We report on the development and validation of an early algorithm to identify which people prescribed TDF/FTC were likely PrEP users. We conducted 2 independent, methodologically distinct validation studies in different populations and with different gold standard methodologies to evaluate the performance of the algorithm. Our results have implications for the development and assessment of algorithm-based estimates of PrEP use [13-15]. Data on PrEP use developed using this or similar algorithms have been used for diverse public health purposes, including tracking trends in PrEP use [15,16], conducting ecological analyses of associations between PrEP prescriptions and HIV infection outcomes [17], evaluating policies related to PrEP uptake [18], and evaluating progress of jurisdictions toward programmatic targets of PrEP uptake [18]. We report the data on this historical algorithm to document the development of what later reports referred to as the “Gilead algorithm” [19]. Presenting these data help complete the documentation of iterative process by which current algorithms have been developed. Its crucial contribution was to propose an algorithm that started with an inclusive list of patients receiving TDF/FTC and to then remove patients with other plausible indications. This general framework is still in use today for PrEP agents that can also be used as therapy for HIV infection or hepatitis B infection.

An evaluation of a similar algorithm was published using data from a single medical center in New York, comparing 3 algorithms in that setting; the authors noted that the publication of such assessments was important [19]. The previous evaluation found high sensitivity and specificity of this algorithm (identified in that paper as the “Gilead algorithm”) in the setting of a large health care provider in New York [19]. Importantly, the performance of an algorithm such as that described here, and the alternative algorithms described by Furukawa et al [19], is that they depend on the quality of information available through medical records. Our validation extends other published evaluations by applying this algorithm in medical settings a different urban area; in a clinic setting more focused on sexual health and HIV prevention and care; and to a national EMR data source, which is more like what is being used in practice to produce estimates of PrEP use. Furthermore, the predictive value of PrEP classifications will vary according to the prevalence of the condition; our report also provides predictive value of negative and positive tests in different geographic settings than the New York study. Indeed, our estimates of PPV and NPV were somewhat different than the application of the “Gilead algorithm” by Furukawa et al [19].

Our results indicated that the PrEP algorithms both had high sensitivity but had more modest and more variable specificity compared with gold standard indications of PrEP use. The modest and variable specificity of the algorithm as measured in the 2 evaluations was somewhat surprising, because the algorithm was constructed to be an approach of exclusion. In this setting, any patient record with an indication of at least 1 competing indication for TDF/FTC prescription was considered not to be a PrEP user; however, both approaches had substantial proportions of false positive results. The sensitivity of the algorithm (both 99%) was higher than the specificity in both evaluations (50% and 87%), and in both settings, the number of PrEP users suggested by the algorithms was higher than the actual number of PrEP users.

The high sensitivity of the algorithm also suggests that estimates of PrEP use made using the algorithm should be considered as highly inclusive estimates of TDF/FTC use for PrEP, because nearly all true PrEP prescriptions are classified as PrEP indications by the algorithm. However, the results of the evaluation in the 2 different settings suggest that the algorithm performance might result in information bias for the national estimates of PrEP use [20], for both algorithms. In both algorithms, there were some misclassifications in both directions, but the number of misclassified cases indicating PrEP uses when TDF/FTC was indicated for other purposes was substantial. Thus, both algorithms substantially overestimated PrEP users, and the predictive value of algorithm-identified PrEP use was low, especially for the EMR study. Even in the case of the clinic-based study, for which specificity was quite high, the high specificity was applied to a much larger pool of non-PrEP users, resulting in substantial numbers of false positive results. This latter scenario is likely most akin to the application of such algorithms to large commercial pharmacy databases [21]. We believe that the applications of the algorithm to national prescription datasets will result in performance more like the EMR validation set, rather than the community clinic dataset, because the national prescription data reflects the prescribing patterns across a range of practice types like the commercial EMR data used in that validation. In any case, sensitivity analyses should be considered for ongoing national estimates of PrEP prescription to characterize uncertainty in estimates attributable to algorithm performance, and the algorithm performance characteristics we report here can be used for that purpose. Because we have reported the performance of the algorithm, including CIs, plausible ranges of PrEP users could be calculated when needed.

Our study had important strengths and limitations. In terms of strengths, both studies used rigorous methods to identify gold standard data on PrEP use. Reviews were comprehensive, considering multiple aspects of medical records, and record reviews were conducted independently by 2 clinician investigators (RMG and SB). Two different approaches and clinical settings were used. The first study used a gold standard list created by the investigators by reviewing each subject using a graphical patient profile. The second study used administrative records from within the clinic to classify patients as PrEP users; by using a smaller, highly circumscribed patient population where the authors knew that PrEP was being administered, a “gold standard” disposition was available for all patient records (vs a small sample in the EMR study, since that required new data collection and decision making on PrEP status for each record).

Despite these strengths, our study also had important weaknesses. Our gold standard data were subject to information bias through misclassification; for example, in the first EMR-based study, the clinical reviewers might have had incomplete or inaccurate data on diagnoses or procedures that led them to incorrectly classify a non-PrEP user as a PrEP user (eg, missing HBV [hepatitis B virus] diagnosis), or to classify a PrEP user as a non-PrEP user (eg, mistakenly coded chronic HBV diagnosis). The EMR data source was likely limited by the missingness of some data to exclude non-PrEP indications for TDF/FTC prescription. For example, some patients identified as PrEP users might have been identified on chart review as false positives for PrEP use because they were patients in care for HIV who had incomplete medication or diagnosis data in their medical records. For the clinic study, it is possible that PrEP user status was misclassified (eg, a patient who was on PrEP but was subsequently diagnosed with HIV infection). Similarly, the algorithm relied on relevant codes to classify indications, and missing or miscoded data could have resulted in incorrect categorization in either direction. Miscoding can be substantial [22], including for infectious viral hepatitis outcomes [16,18,23-27]. The relative performance of the algorithms in the 2 evaluations was also affected by the types of data included in the datasets. The EMR validation study had a population that comprised a larger proportion of patients with HIV who were easily and correctly classified; the community health clinic study had a lower ratio of PrEP to patients with HIV because of the nature of the programs offered in the clinic. The data were from 2012 to 2014, and current prescribing practices have likely changed. During the period of the evaluation (specifically the early part of 2012), TDF/FTC was not approved for PrEP use, and off-label use would have lacked appropriate Centers for Medicaid and Medicare Services coding. This research was conducted with *ICD-9* codes, which were the most current at the time of the research; confirmation of validation and remapping to *ICD-10* codes will be required.

The results presented here are historical, and prescription practices have almost certainly evolved since the data were collected. Despite this, significant aspects of the methods used here are currently in use by both Centers for Disease Control and Prevention [28] and AIDSVu [29] for production of national estimates of PrEP use. These data are being used widely for public health relevant analyses of PrEP [30], and we therefore believe it is important to have a peer-reviewed, transparent documentation of these methods. We recognize that both the medications used for PrEP and the prescriptions patterns issued for PrEP have evolved since this work was conducted and will continue to evolve in the future [28]. Specifically, we recognize the need to develop improved methods to understand how to characterize the use of TDF/FTC as event-driven PrEP [29], to incorporate new formulations of PrEP [30], and to prepare for increased use of long-acting injectable PrEP [31]. There is a need to update the *ICD-9* codes used in this evaluation to contemporary *ICD-10* codes. To date, we have implemented further changes to the algorithm and provided notes as data methods on the AIDSVu website to account for changes in agents and PrEP guidelines, such as the addition of emtricitabine and tenofovir alafenamide [30] and injectable cabotegravir [31,32]. The AIDSVu link will be maintained as a live repository of current methods used for AIDSVu public use datasets. Given the increasing complexity of agents and prescribing patterns, we identify the need for resources and a consensus and living approach to developing a shared case definition for PrEP use for public health monitoring purposes and tracking of progress toward national prevention priorities.

The algorithm described here is primarily of use for monitoring of state and national progress toward prevention goals using data from national prescription databases [21,22,33]. Clinics might have access to additional clinical data that would help improve such an algorithm for assessment of clinic progress in improving PrEP programs.

Validation studies conducted with 2 different methodologies demonstrate that an algorithm of exclusion using *ICD-9* codes, procedure codes, and concomitantly prescribed medications can meaningfully identify PrEP indications in medical claims databases and EMRs, with some defined occurrence of misclassification. The validation provides an important bridge between studies of PrEP uptake and the ability to evaluate PrEP use using real-world data. We recommend that public health researchers using algorithms to estimate numbers of PrEP users conduct sensitivity analyses using the performance data from these studies to evaluate the impact of algorithm-based classification in specific clinical or data settings. Given the complexities of this task, machine learning could be considered for ongoing evaluation of the best ways to monitor PrEP use; such methods have already been applied to identifying potential PrEP indications [34-37]. Whatever methods are used now or in the future, validations should be updated as prescribing patterns change, for new PrEP formulations already available [30], and as new options for PrEP come onto the market [38].

## Abbreviations

CDC: Centers for Disease Control and Prevention
CHB: chronic hepatitis B
EMR: electronic medical record
FDA: Food and Drug Administration
HBV: hepatitis B virus
HIPAA: Health Insurance Portability and Accountability Act
ICD: International Classification of Diseases
IRB: institutional review board
NIH: National Institutes of Health
NPV: negative predictive value
PEP: postexposure prophylaxis
PPV: positive predictive value
PrEP: pre-exposure prophylaxis
TDF/FTC: tenofovir disoproxil fumarate and emtricitabine

## Data Availability

The datasets generated during and/or analyzed during this study are not publicly available because they represent individual data from patient care and are protected by HIPAA (Health Insurance Portability and Accountability Act). Data from the sexual health clinic records were abstracted by study personnel with institutional review board (IRB) approval, and individual medical records or abstracted data are not available to personnel not included in the study IRB. We will make data collection forms and protocols available on request, should others wish to evaluate similar data from other patient populations; such data collections would presumably require IRB review by the relevant institutions.
